# Conduction system vs. biventricular pacing in heart failure with reduced ejection fraction: a meta-analysis of randomized controlled trials

**DOI:** 10.1093/europace/euag170

**Published:** 2026-08-13

**Authors:** Paschalis Karakasis, Konstantinos Vlachos, Konstantinos C Siontis, Óscar Cano, Stylianos Tzeis, Marc Strik, Konstantinos Pamporis, Georgios Leventopoulos, Antonios P Antoniadis, Pierre Jaïs, Haran Burri, Nikolaos Fragakis

**Affiliations:** Second Department of Cardiology, Hippokration General Hospital, Aristotle University of Thessaloniki, Thessaloniki, Greece; Electrophysiology and Heart Modeling Institute, IHU Liryc, INSERM U1045, Fondation Bordeaux Université and Université de Bordeaux, Pessac, France; Cardiac Arrhythmia Department, INSERM U1045, CHU de Bordeaux, Pessac, France; Electrophysiology Department, Hygeia Hospital, Athens, Greece; Department of Cardiovascular Medicine, Mayo Clinic, Rochester, MN, USA; Department of Cardiology, Hospital Universitario y Politécnico La Fe, Planta 4-Torre F, Av. Fernando Abril Martorell, 106, Valencia 46026, Spain; Department of Cardiology, Mitera Hospital, 6, Erythrou Stavrou Str. 151 23, Marousi, Athens, Greece; Electrophysiology and Heart Modeling Institute, IHU Liryc, INSERM U1045, Fondation Bordeaux Université and Université de Bordeaux, Pessac, France; Cardio-Thoracic Unit, Bordeaux University Hospital (CHU), Pessac, France; First Department of Cardiology, School of Medicine, National and Kapodistrian University of Athens, Hippokration General Hospital, Athens, Greece; Department of Cardiology, University Hospital of Patras, Patras 26504, Greece; Department of Cardiology, University Hospital Southampton NHS Foundation Trust, Southampton, UK; Second Department of Cardiology, Hippokration General Hospital, Aristotle University of Thessaloniki, Thessaloniki, Greece; Electrophysiology and Heart Modeling Institute, IHU Liryc, INSERM U1045, Fondation Bordeaux Université and Université de Bordeaux, Pessac, France; Cardiac Arrhythmia Department, INSERM U1045, CHU de Bordeaux, Pessac, France; Cardiac Pacing Unit, Department of Cardiology, University Hospital of Geneva, Geneva, Switzerland; Second Department of Cardiology, Hippokration General Hospital, Aristotle University of Thessaloniki, Thessaloniki, Greece

**Keywords:** Conduction system pacing, Biventricular pacing, Cardiac resynchronization therapy, Heart failure with reduced ejection fraction, Left bundle branch area pacing, Hospitalization for heart failure, Mortality

## Abstract

**Aims:**

Conduction system pacing (CSP) has emerged as a physiologic alternative to cardiac resynchronization therapy (CRT) with biventricular pacing (BiVP) in heart failure with reduced ejection fraction (HFrEF), yet its comparative effects remain uncertain. This meta-analysis sought to compare outcomes of CSP vs. BiVP in patients with HFrEF undergoing CRT.

**Methods and results:**

MEDLINE, Embase, Scopus, and the Cochrane Database were searched through 4 May 2026. Randomized controlled trials (RCTs) were synthesized using random-effects meta-analysis. Thirteen RCTs including 1320 participants were analysed (657 CSP, 663 BiVP). Conduction system pacing was associated with a lower risk of the composite of hospitalization for heart failure (HHF) or all-cause mortality compared with BiVP (46 vs. 83 events; odds ratio [OR] 0.51, 95% confidence interval [CI] 0.28–0.93). No significant differences were observed for HHF alone (OR 0.68, 95% CI 0.36–1.27) or all-cause mortality (OR 0.75, 95% CI 0.36–1.58). Conduction system pacing was associated with greater improvement in the 6-min walk distance (mean difference [MD] 21.53 m, 95% CI 4.33–38.73) and New York Heart Association functional class (standardized mean difference [SMD] −0.27, 95% CI −0.47 to −0.07), but not quality of life. Conduction system pacing also reduced QRS duration compared with BiVP (MD −10.94 ms, 95% CI −17.55 to −4.34), with no significant differences in left ventricular ejection fraction, left ventricular end-systolic volume, overall pacing threshold, total procedure time, fluoroscopy time, any complication, or reintervention.

**Conclusion:**

In patients with HFrEF undergoing CRT, CSP is associated with lower composite risk of HHF or all-cause mortality compared with BiVP. Conduction system pacing is also associated with improved functional capacity, without significant differences in safety or procedural outcomes.

## Graphical Abstract

**Graphical Abstract:**
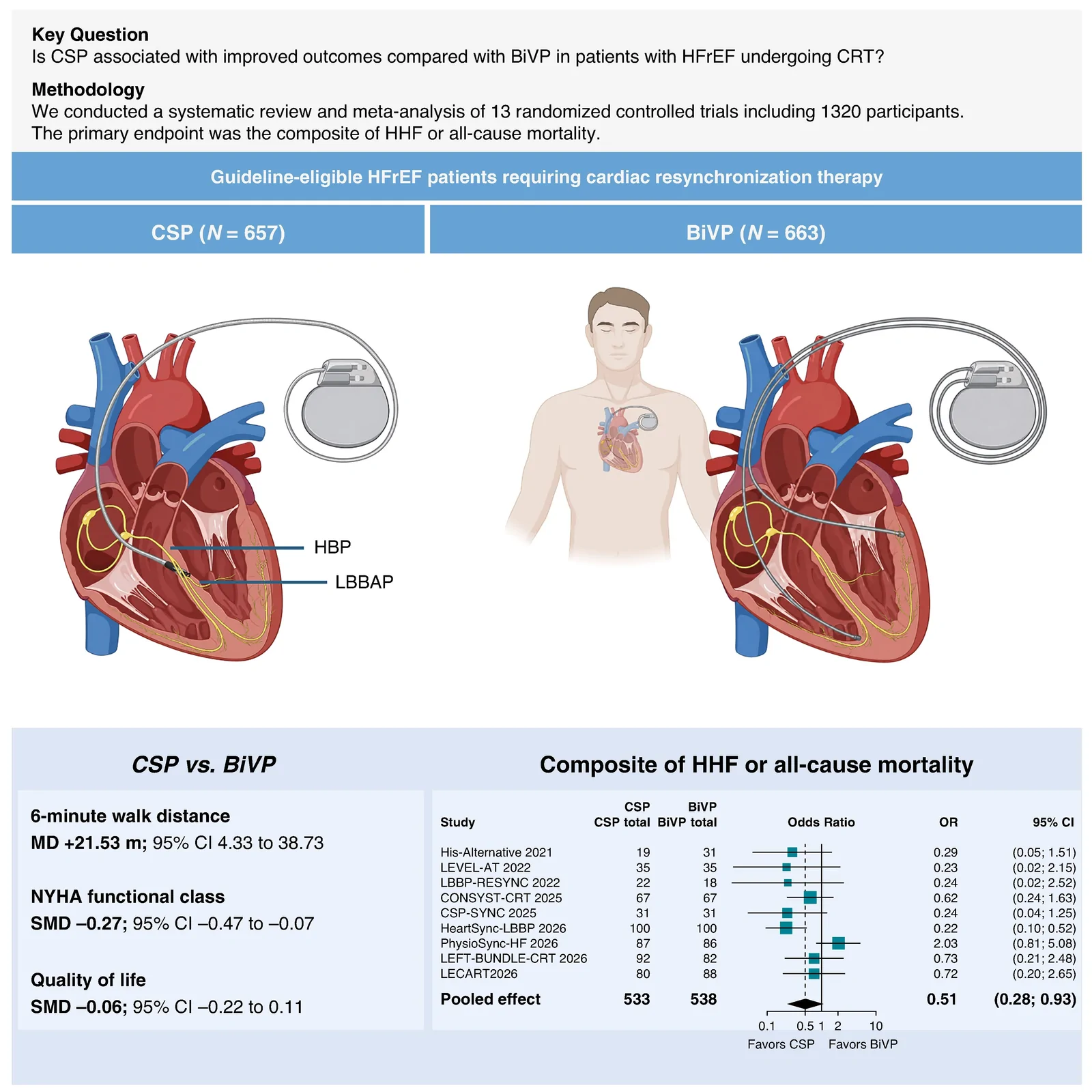

## Introduction

Cardiac resynchronization therapy (CRT) is an established treatment for selected patients with heart failure with reduced ejection fraction (HFrEF) and electrical dyssynchrony, improving symptoms, attenuating adverse remodelling, and reducing hospitalization for heart failure (HHF) and mortality.^1–6^ Conventional CRT delivered through biventricular pacing (BiVP) has therefore become a foundational therapy in this population. However, BiVP restores synchrony indirectly through epicardial left ventricular (LV) activation and may provide incomplete electrical correction when coronary venous anatomy, LV lead position, myocardial scar, or residual conduction delay limit effective resynchronization.^7,8^

Conduction system pacing (CSP) has emerged as a more physiological pacing strategy, designed to reproduce native His–Purkinje-mediated ventricular activation and correct conduction delay through recruitment of the intrinsic cardiac conduction system.^9–11^ Whereas early randomized experience focused largely on His bundle pacing (HBP), more recent trials have increasingly evaluated left bundle branch area pacing (LBBAP), reflecting the procedural evolution of CSP towards approaches with lower pacing thresholds and potentially more stable electrical performance.^12–16^ Across these studies, CSP has generally shown favourable effects on electrical resynchronization, functional status, and selected indices of reverse remodelling. However, the randomized evidence base consists of modest-sized trials with heterogeneous pacing strategies and follow-up durations.

The key knowledge gap is whether the physiological rationale of CSP translates into fewer clinically consequential events. Existing randomized trials were not individually powered to assess HHF or mortality, and most were designed around surrogate, functional, or remodelling endpoints. Consequently, the comparative effect of CSP vs. BiVP on hard clinical outcomes remains uncertain, despite the rapid expansion of CSP in contemporary CRT practice.^17,18^

Accordingly, we performed a systematic review and meta-analysis of randomized controlled trials (RCTs) comparing CSP with BiVP in patients with HFrEF undergoing CRT.

## Methods

This systematic review and meta-analysis was designed and conducted in accordance with the Cochrane Handbook for Systematic Reviews^19^ and reported in compliance with the PRISMA 2020 statement.^20^ The protocol was prospectively registered in the Open Science Framework (doi: https://doi.org/10.17605/OSF.IO/K3J2U), and the prespecified analytical framework was followed without deviation. Because the study used only aggregate data from previously published reports, ethics committee approval and informed consent were not required.

### Search strategy

The search strategy was developed a priori by three investigators. MEDLINE via PubMed, Embase, Scopus, and the Cochrane Library/CENTRAL were searched from database inception to 4 May 2026, without restrictions on language, publication year, publication status, or dissemination date. The search syntax combined controlled vocabulary and free-text terms related to CSP, HBP, left bundle branch pacing, LBBAP, BiVP, CRT, heart failure, and randomized trials. To maximize retrieval sensitivity, supplementary searches were performed in ClinicalTrials.gov, Epistemonikos, and Google Scholar, and backward and forward citation tracking was undertaken using the citationchaser package in R.^21^ The complete electronic search strategies are provided in Supplementary material online, *Tables S1*–*S4*.

### Eligibility criteria

#### Inclusion criteria

Eligible studies were RCTs enrolling adults with HFrEF who underwent CRT and directly compared a CSP strategy with BiVP. CSP strategies included HBP, LBBAP, or mixed CSP approaches. Trials were eligible if they reported at least one prespecified outcome.

#### Exclusion criteria

The following were excluded: non-comparative reports, including case reports, case series, and narrative reviews; non-original publications, including editorials, letters, commentaries, and expert opinion articles; study protocols, clinical practice guidelines, and unpublished theses or dissertations. Observational studies and nonrandomized interventional studies were also excluded.

### Outcomes

The primary endpoint was the composite of HHF or all-cause mortality, while HHF and all-cause mortality were also analysed separately as individual secondary clinical outcomes. Functional and patient-centred outcomes comprised the 6-min walk distance, New York Heart Association (NYHA) functional class, and quality-of-life (QoL) measures, assessed using the Minnesota Living with Heart Failure Questionnaire, Kansas City Cardiomyopathy Questionnaire, EQ-5D score, and EuroQol visual analogue scale. Electrocardiographic and echocardiographic outcomes included QRS duration, LV ejection fraction (LVEF), and LV end-systolic volume (LVESV). Procedural and device performance outcomes included total procedure time, fluoroscopy time, and pacing threshold. Safety outcomes comprised any complication and reintervention. All outcomes were extracted according to trial-level definitions.

### Study selection

Study selection was performed independently by three investigators using a two-stage process. In the first stage, all retrieved records were screened by title and abstract, and any record considered potentially eligible by at least one reviewer was advanced to full-text assessment. In the second stage, full-text reports were independently evaluated against the prespecified eligibility criteria to determine final inclusion. Disagreements were resolved through discussion and consensus, with adjudication by a senior author when necessary. Screening was conducted in Abstrackr,^22^ while citation management and duplicate removal were performed in Mendeley.

### Data extraction

A standardized data extraction form was developed a priori and refined through pilot testing before formal data collection. Data extraction was performed independently by three investigators, with discrepancies resolved by consensus and, when required, adjudication by a senior author.

Extracted information included trial characteristics, including first author, year of publication, country, enrolment period, study design, sample size, follow-up duration, intervention and control strategies, CSP approach, and device/procedural characteristics. Baseline participant-level characteristics included age, sex, coronary artery disease, NYHA functional class, LVEF, QRS duration, and other relevant clinical variables. Outcome-level data included effect estimates with corresponding 95% confidence intervals (CIs), and raw event counts or continuous outcome data, as applicable. When required, additional information was sought from the corresponding authors of the original reports.

### Quality assessment

The methodological rigour of the included RCTs was appraised using the revised Cochrane Risk of Bias tool (RoB 2).^23^ Three reviewers independently assessed each trial across the five prespecified domains: bias arising from the randomization process, deviations from intended interventions, missing outcome data, outcome measurement, and selection of the reported result. Overall risk-of-bias judgments were assigned according to RoB 2 guidance, with a trial classified as low risk only when all component domains were judged to be at low risk. Any discrepancies were resolved through consensus, with arbitration by a senior reviewer when necessary

### Data analysis

All statistical analyses were performed in R version 4.5.3. Dichotomous outcomes were synthesized as odds ratios (ORs) with 95% CIs, whereas continuous outcomes were pooled as mean differences (MDs) or standardized mean differences (SMDs). Separate multilevel random-effects meta-analyses were performed for each prespecified outcome. Between-study variance was estimated within a frequentist framework using restricted maximum likelihood. Statistical significance was defined by a two-sided *P* value <0.05.

Between-study heterogeneity was quantified using the *I*^2^ statistic and assessed formally with Cochran's *Q* test. *I*^2^ values of 0–30%, 30–50%, 50–75%, and 75–100% were interpreted as low, moderate, substantial, and considerable heterogeneity, respectively.^24^

Prespecified subgroup analyses were performed according to CSP approach, comparing HBP with LBBP/LBBAP strategies, with subgroup differences evaluated using interaction testing. Influence diagnostics were used to identify studies exerting disproportionate influence on the pooled effect estimate or between-study heterogeneity, including Baujat plots and standard influence analyses. Leave-one-out sensitivity analyses were additionally performed by iteratively omitting each study from the pooled model to evaluate the robustness of the summary estimate.

In accordance with Cochrane recommendations, small-study effects and publication bias were assessed with contour-enhanced funnel plots and Egger's regression test only when at least 10 studies contributed to an outcome.^25^

### Certainty of evidence assessment

The certainty of evidence was appraised for the outcomes considered most critical for clinical interpretation and decision-making, in accordance with Cochrane guidance recommending that Summary of Findings tables prioritize a limited set of critical or important outcomes.^26^ These outcomes were the composite of HHF or all-cause mortality, HHF, all-cause mortality, 6-min walk distance, NYHA functional class, and QoL. Judgments were made across the core GRADE domains, including risk of bias, inconsistency, indirectness, imprecision, and publication bias, and disagreements were resolved through discussion and, when necessary, adjudication by the senior reviewer. Certainty ratings and the Summary of Findings table were generated using the GRADEpro Guideline Development Tool (GRADEpro GDT; McMaster University and Evidence Prime, 2024).

## Results

### Study selection and characteristics

The study selection process is presented in the PRISMA 2020 flow diagram (see Supplementary material online, *Figure S1*). Overall, 13 RCTs were included,^13–15,27–36^ with additional long-term follow-up extension reports available for the His-Alternative^37^ and LBBP-RESYNC^38^ trials. The randomized evidence base comprised 1320 participants, including 657 patients assigned to a CSP strategy and 663 assigned to BiVP. The intervention consisted of HBP in four trials, LBBP/LBBAP in five trials, and mixed CSP approaches in four trials, whereas BiVP constituted the control strategy in all included comparisons.

The key characteristics of the included trials are summarized in *Table 1*. Across the primary trial reports, follow-up ranged from 6 to 36 months, with a median duration of 12 months (interquartile range, 12–18 months). Mean age ranged from 61.0 to 71.3 years, and the proportion of male participants ranged from 35% to 90% across treatment groups. Mean LVEF ranged from 26.5% to 34.0%, while baseline QRS duration ranged from 99.4 to 184 ms.

**Table 1:** Characteristics of included randomized trials comparing conduction system pacing with biventricular pacing

Study, year	Intervention vs. control	Follow-up, months	*n* (I/C)	Age, years (I/C)	Male, % (I/C)	CAD, % (I/C)	NYHA II, % or mean (SD) (I/C)	NYHA III, % (I/C)	LVEF, % mean (SD) (I/C)	QRS, ms mean (SD) (I/C)	His pacing, %	LBBP, %	LBBAP, %
**Lustgarten et al., 2015^30^**	HBP vs. BiVP	6	29/29	71.3 (24)	66.6	55	7	86	26.5 (8.7)	162 (45)	72	0	0
**His-SYNC, 2019^35^**	HBP vs. BiVP	12	16/24	63.4 (13.3)/65.5 (12.4)	56/66	63/67	2.7 (0.6)/2.7 (0.6)	NR	28.4 (8.9)/27.3 (5.6)	174 (18)/165 (17)	52	0	0
**His-Alternative, 2021^14^**	HBP vs. BiVP	6 (ext. 63.6)	25/25	63.8 (9.4)/67.7 (9)	56/72	20/24	2.4 (0.4)/2.4 (0.4)	NR	30 (8)/30 (6)	167 (16)/165 (14)	72	0	0
**LEVEL-AT, 2022^32^**	HBP/LBBP vs. BiVP	6	35/35	65.7 (9)/68.1 (9)	88/90	11/11	54.3/48.6	31.4/37.1	27 (7)/28 (7)	177 (21)/178 (22)	11	66	66
**LBBB-RESYNC, 2022^33^**	LBBP vs. BiVP	6 (ext. 48)	20/20	62.3 (11.2)/65.3 (10.6)	35/65	0/0	2.4 (0.5)/2.45 (0.51)	NR	28.3 (5.3)/31.1 (5.6)	174.6 (14.3)/174.7 (14.1)	0	90	90
**ALTERNATIVE-AF, 2022^28^**	HBP + AVNA vs. BiVP + AVNA	18	50 (25 in each group)	63.6 (12.1)/65 (8.2)	76/68	24/24	2.8 (0.6)/2.8 (0.5)	NR	31.9 (7)/34 (4.8)	99.8 (15.3)/99.4 (13.6)	100	0	0
**HOT-CRT, 2023^36^**	HBP/LBBAP vs. BiVP	6	50/50	71 (13.4)/68.9 (11.5)	78/60	46/44	2.5 (0.7)/2.5 (0.7)	NR	30.1 (9.1)/30.7 (9.3)	164 (26)/166 (28)	8	NR	88
**CONSYST-CRT, 2025^27^**	HBP/LBBP vs. BiVP	12	67/67	69 (9)/69 (9)	72/75	34/34	59.7/47.8	34.3 (NYHA III–IV)/44.8 (NYHA III–IV)	28 (6)/27 (7)	175 (22)/175 (23)	6	67	67
**CSP-SYNC, 2025^34^**	LBBAP vs. BiVP	12	31/31	65.4 (10.1)/67.9 (15.5)	74/67	35/29	77/68	23/32	30 (5)/28 (6)	176 (18)/170 (19)	0	90	97
**LEFT-BUNDLE CRT, 2026^15^**	LBBAP vs. BiVP	12	92/83	67.4 (9.8)/68.7 (12.1)	66/68	27/28	76/65	13/27	30 (7.5)/29.4 (6.8)	171 (19.6)/165.7 (21)	0	NR	90
**PhysioSync-HF, 2026^31^**	LBBAP/HBP vs. BiVP	12	87/86	61 (9)/63.3 (9.8)	48/52	15/11	54/52	45/48	26.7 (6.4)/26.6 (7.4)	184 (23)/184 (23)	2	NR	71
**HeartSync-LBBP, 2026^13^**	LBBP vs. BiVP	36 (median)	100/100	64.3 (9.5)/65.3 (9.5)	67/69	16/19	2.9 (0.5)/3 (0.6)	NR	28.3 (3.8)/28.1 (5)	169.8 (19)/167 (18)	0	98	98
**LECART, 2026^29^**	LBBAP vs. BiVP	12	80/88	69	67	20	67	NR	30.5	NR	0	NR	94

AVNA, atrioventricular node ablation; BiVP, biventricular pacing; CAD, coronary artery disease; C, control; ext., trial extension; HBP, His bundle pacing; I, intervention; LBBAP, left bundle branch area pacing; LBBP, left bundle branch pacing; LVEF, left ventricular ejection fraction; NR, not reported; NYHA, New York Heart Association; QRS, QRS complex duration; SD, standard deviation.

Risk-of-bias assessments for the included RCTs are shown in Supplementary material online, *Figure S2*. Most trials were judged to be at low risk of bias overall (8/13), while the remaining trials were rated as having some concerns (5/13).

### Composite of hospitalization for heart failure or all-cause mortality

**Figure 1:**
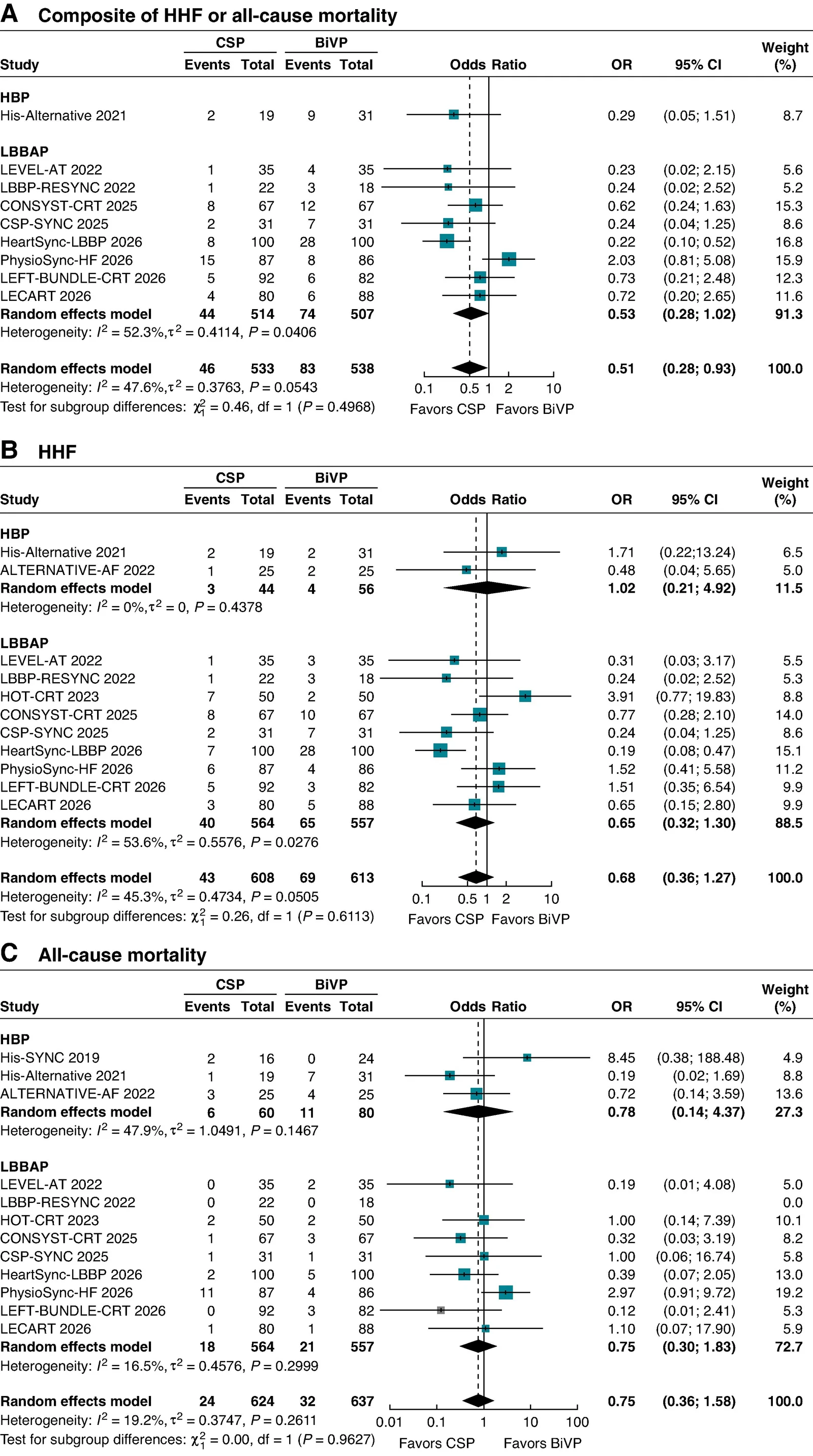
Forest plots showing the comparative effect of CSP vs. BiVP on (*A*) the composite of HHF or all-cause mortality, (*B*) HHF, and (*C*) all-cause mortality. Effect estimates are presented as ORs with 95% CIs, pooled using random-effects models. Squares represent study-specific ORs, with square size proportional to study weight; horizontal lines indicate 95% CIs. Diamonds represent pooled estimates for each subgroup and for the overall analysis. Analyses were stratified according to CSP approach, including HBP and LBBAP. Values <1 favour CSP, whereas values >1 favour BiVP. BiVP, biventricular pacing; CI, confidence interval; CSP, conduction system pacing; HBP, His bundle pacing; HHF, hospitalization for heart failure; LBBAP, left bundle branch area pacing; OR, odds ratio.

Nine RCTs contributed to the analysis of the composite endpoint of HHF or all-cause mortality, representing 1071 participants (533 assigned to CSP and 538 to BiVP). Overall, 129 events were recorded, with 46 events in the CSP group and 83 in the BiVP group. Conduction system pacing was associated with a significantly lower risk of the composite endpoint compared with BiVP (OR 0.51, 95% CI 0.28–0.93, *P* = 0.028; *I*^2^ = 47.6%, *P* for heterogeneity = 0.054; *Figure 1A*). The treatment effect was directionally consistent across CSP subgroups, with no evidence of a subgroup difference between HBP and LBBAP (*P* for subgroup difference = 0.50).

### Hospitalization for heart failure

Eleven RCTs contributed to the analysis of HHF, representing 1221 participants (608 assigned to CSP and 613 to BiVP). Overall, 112 events were recorded, with 43 events in the CSP group and 69 in the BiVP group. There was no statistically significant difference between treatment strategies (OR 0.68, 95% CI 0.36–1.27, *P* = 0.23; *I*^2^ = 45.3%, *P* for heterogeneity = 0.051; *Figure 1B*). No significant subgroup difference was observed between HBP and LBBAP (*P* for subgroup difference = 0.61).

### All-cause mortality

Twelve RCTs contributed to the analysis of all-cause mortality, representing 1261 participants (624 assigned to CSP and 637 to BiVP). Overall, 56 deaths were recorded, with 24 deaths in the CSP group and 32 in the BiVP group. There was no significant difference between CSP and BiVP for all-cause mortality (OR 0.75, 95% CI 0.36–1.58, *P* = 0.45; *I*^2^ = 19.2%, *P* for heterogeneity = 0.26; *Figure 1C*). Treatment effects were similar across HBP and LBBAP subgroups, with no evidence of a subgroup difference (*P* for subgroup difference = 0.96).

### 6-Min walk distance

**Figure 2:**
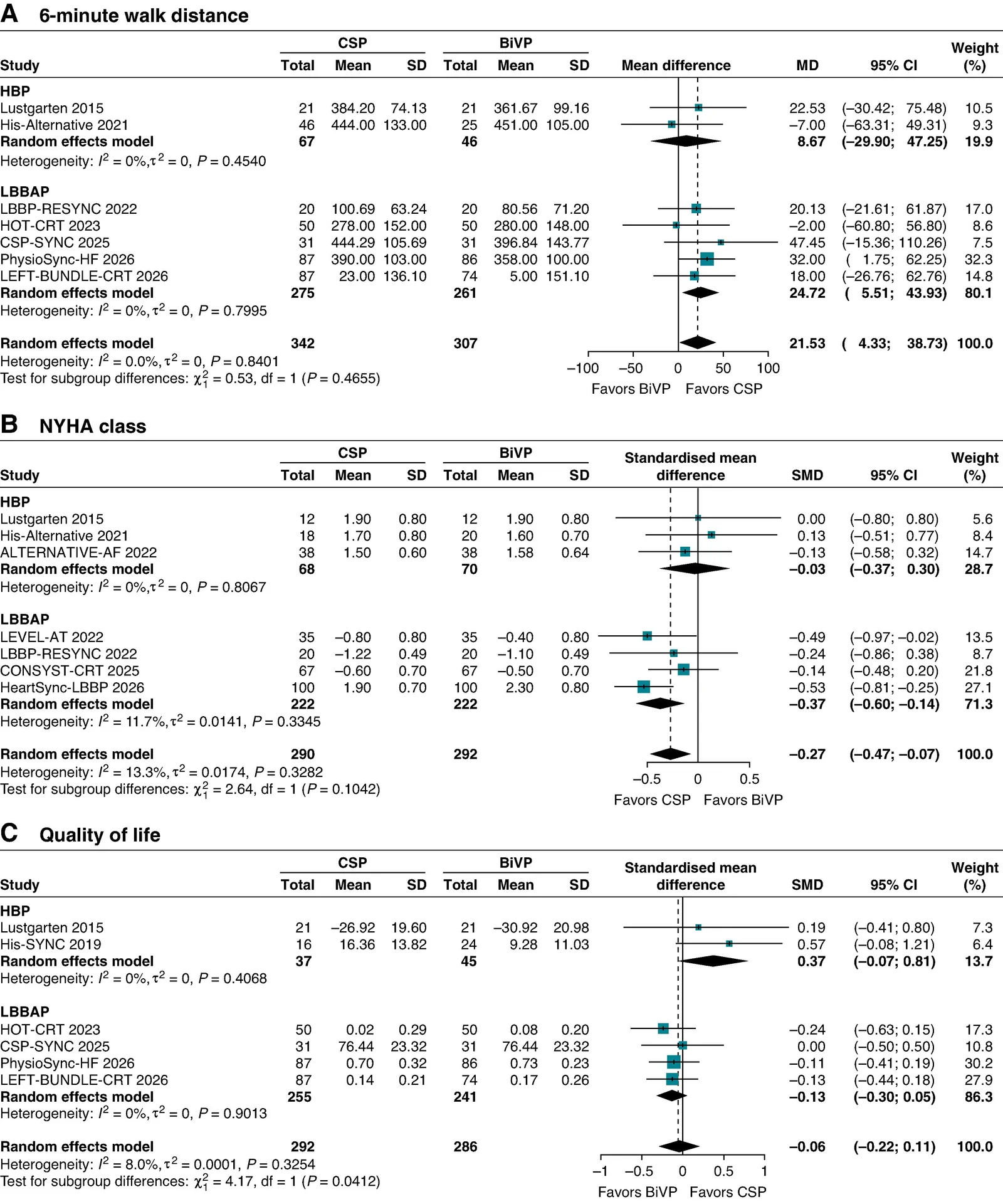
Forest plots showing the comparative effect of CSP vs. BiVP on (*A*) 6-min walk distance, (*B*) NYHA functional class, and (*C*) quality of life. Effect estimates are presented as MDs for 6-min walk distance and SMDs for NYHA class and quality-of-life outcomes, with corresponding 95% CIs. Analyses were stratified according to CSP approach, including HBP and LBBAP. BiVP, biventricular pacing; CI, confidence interval; CSP, conduction system pacing; HBP, His bundle pacing; LBBAP, left bundle branch area pacing; MN, mean difference; NYHA, New York Heart Association; SMD, standardized mean difference.

Seven RCTs contributed to the analysis of 6-min walk distance, representing 649 participants (342 assigned to CSP and 307 to BiVP). Conduction system pacing was associated with a significantly greater improvement in 6-min walk distance compared with BiVP (MD 21.53 m, 95% CI 4.33–38.73, *P* = 0.014; *I*^2^ = 0.0%, *P* for heterogeneity = 0.84; *Figure 2A*). There was no evidence of a subgroup difference between HBP and LBBAP (*P* for subgroup difference = 0.47).

### New York Heart Association functional class

Seven RCTs contributed to the analysis of NYHA functional class, representing 582 participants (290 assigned to CSP and 292 to BiVP). Conduction system pacing was associated with a significantly greater improvement in NYHA class compared with BiVP (SMD −0.27, 95% CI −0.47 to −0.07, *P* = 0.008; *I*^2^ = 13.3%, *P* for heterogeneity = 0.33; *Figure 2B*). There was no evidence of a subgroup difference between HBP and LBBAP (*P* for subgroup difference = 0.10).

### Quality of life

Six RCTs contributed to the analysis of QoL outcomes, representing 578 participants (292 assigned to CSP and 286 to BiVP). There was no significant difference between CSP and BiVP in QoL measures (SMD −0.06, 95% CI −0.22 to 0.11, *P* = 0.48; *I*^2^ = 8.0%, *P* for heterogeneity = 0.37; *Figure 2C*). In subgroup analyses, the pooled estimates were not significant in either the HBP subgroup (SMD 0.37, 95% CI −0.07 to 0.81) or the LBBAP subgroup (SMD −0.13, 95% CI −0.30 to 0.05). A significant subgroup difference was observed between HBP and LBBAP (*P* for subgroup difference = 0.041).

### QRS duration

**Figure 3:**
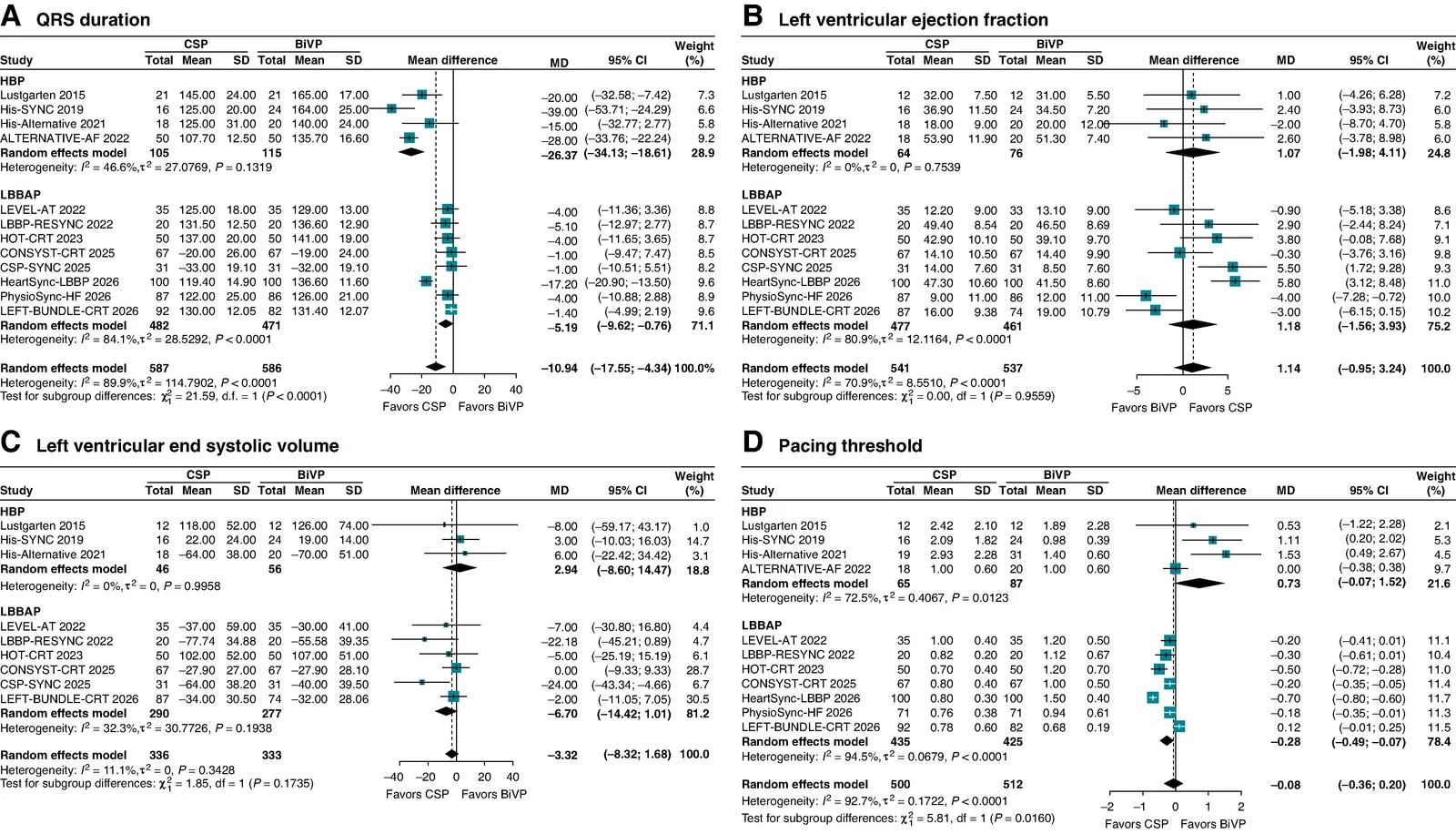
Forest plots showing the comparative effect of CSP vs. BiVP on (*A*) QRS duration, (*B*) left ventricular ejection fraction, (*C*) left ventricular end-systolic volume, and (*D*) pacing threshold. Effect estimates are presented as MDs with 95% CIs, pooled using random-effects models. Analyses were stratified according to CSP approach, including HBP and LBBAP. BiVP, biventricular pacing; CI, confidence interval; CSP, conduction system pacing; HBP, His bundle pacing; LBBAP, left bundle branch area pacing; MN, mean difference.

Twelve RCTs contributed to the analysis of QRS duration, representing 1173 participants (587 assigned to CSP and 586 to BiVP). CSP was associated with a significantly shorter QRS duration compared with BiVP (MD −10.94 ms, 95% CI −17.55 to −4.34, *P* = 0.001; *I*^2^ = 89.9%, *P* for heterogeneity < 0.001; *Figure 3A*). In subgroup analyses, significant reductions in QRS duration were observed in both the HBP subgroup (MD −26.37 ms, 95% CI −34.13 to −18.61) and the LBBAP subgroup (MD −5.19 ms, 95% CI −9.62 to −0.76; *P* for subgroup difference < 0.001).

### Left ventricular ejection fraction

Twelve RCTs contributed to the analysis of LVEF, representing 1078 participants (541 assigned to CSP and 537 to BiVP). There was no significant difference between CSP and BiVP in LVEF (MD 1.14 percentage points, 95% CI −0.95 to 3.24, *P* = 0.29; *I*^2^ = 70.9%, *P* for heterogeneity < 0.001; *Figure 3B*). There was no evidence of a subgroup difference between HBP and LBBAP (*P* for subgroup difference = 0.96).

### Left ventricular end-systolic volume

Nine RCTs contributed to the analysis of LVESV, representing 669 participants (336 assigned to CSP and 333 to BiVP). There was no significant difference between CSP and BiVP in LVESV (MD −3.32 mL, 95% CI −8.32 to 1.68, *P* = 0.19; *I*^2^ = 11.1%, *P* for heterogeneity = 0.34; *Figure 3C*). There was no evidence of a subgroup difference between HBP and LBBAP (*P* for subgroup difference = 0.17).

### Pacing threshold

Eleven RCTs contributed to the analysis of pacing threshold, representing 1012 participants (500 assigned to CSP and 512 to BiVP). There was no significant difference between CSP and BiVP in pacing threshold (MD −0.08 V, 95% CI −0.36 to 0.20, *P* = 0.58; *I*^2^ = 92.7%, *P* for heterogeneity < 0.001; *Figure 3D*). In subgroup analyses, the pooled estimate was not significant in the HBP subgroup (MD 0.73 V, 95% CI −0.07 to 1.52), whereas a significant reduction was observed in the LBBAP subgroup (MD −0.28 V, 95% CI −0.49 to −0.07; *P* for subgroup difference = 0.016).

### Any complication

Ten RCTs contributed to the analysis of any complication, representing 957 participants (479 assigned to CSP and 478 to BiVP). Overall, 67 events were recorded, with 29 events in the CSP group and 38 in the BiVP group. There was no significant difference between CSP and BiVP for any complication (OR 0.78, 95% CI 0.45–1.35, *P* = 0.38; *I*^2^ = 0.0%, *P* for heterogeneity = 0.68; Supplementary material online, *Figure S3A*). There was no evidence of a subgroup difference between HBP and LBBAP (*P* for subgroup difference = 0.92).

### Reintervention

Seven RCTs contributed to the analysis of reintervention, representing 863 participants (431 assigned to CSP and 432 to BiVP). Overall, 53 events were recorded, with 30 events in the CSP group and 23 in the BiVP group. There was no significant difference between CSP and BiVP for reintervention (OR 1.44, 95% CI 0.54–3.86, *P* = 0.47; *I*^2^ = 54.4%, *P* for heterogeneity = 0.04; Supplementary material online, *Figure S3B*). In subgroup analyses, the individual HBP study showed a higher risk of reintervention (OR 12.83, 95% CI 2.87–57.46), whereas the pooled estimate in the LBBAP subgroup was not significant (OR 0.87, 95% CI 0.44–1.69; *P* for subgroup difference = 0.001).

### Total procedure time

Ten RCTs contributed to the analysis of total procedure time, representing 1064 participants (544 assigned to CSP and 520 to BiVP). Overall, there was no significant difference between CSP and BiVP in total procedure time (MD 1.61 min, 95% CI −7.14 to 10.36; *I*^2^ = 64.6%, *P* for heterogeneity = 0.0025; Supplementary material online, *Figure S4*). In subgroup analyses, HBP was associated with significantly longer total procedure time compared with BiVP (MD 34.33 min, 95% CI 16.81–51.85), whereas the pooled estimate in the LBBAP subgroup was not significant (MD −1.67 min, 95% CI −7.11 to 3.76; *P* for subgroup difference = 0.0001).

### Fluoroscopy time

Nine RCTs contributed to the analysis of fluoroscopy time, representing 1003 participants (507 assigned to CSP and 496 to BiVP). There was no significant difference between CSP and BiVP in fluoroscopy time (MD −0.19 min, 95% CI −3.85 to 3.47, *P* = 0.92; *I*^2^ = 82.9%, *P* for heterogeneity < 0.001; Supplementary material online, *Figure S5*). There was no significant subgroup difference between HBP and LBBAP (*P* for subgroup difference = 0.094).

### Sensitivity and influence analyses

For the primary composite endpoint of HHF or all-cause mortality, Baujat (see Supplementary material online, *Figure S6*) and influence diagnostic plots (see Supplementary material online, *Figure S7*) identified PhysioSync-HF 2026 as an outlying and influential study, with the largest contribution to between-study heterogeneity and the pooled treatment estimate. In the leave-one-out analysis, exclusion of this study strengthened the association in favour of CSP and eliminated heterogeneity (OR 0.39, 95% CI 0.24–0.64, *P* < 0.001; *I*^2^ = 0.0%; Supplementary material online, *Figure S8*).

Leave-one-out and influence analyses for the remaining outcomes are presented in Supplementary material online, *Figures S9*–*S47*.

### Small-study effect analyses

No evidence of small-study effects or publication bias was identified based on visual inspection of the contour-enhanced funnel plots and the results of Egger's regression tests (see Supplementary material online, *Figures S48*–*S54*).

### Certainty of evidence

The certainty of evidence for the prespecified outcomes is summarized in Supplementary material online, *Table S6*. Certainty was rated as moderate across all outcomes. Downgrading was attributable to imprecision, reflecting limited event accrual for dichotomous endpoints and CIs that did not exclude clinically important uncertainty in effect magnitude for continuous endpoints.

## Discussion

This meta-analysis assembles the totality of randomized evidence to date—13 RCTs and 1320 participants—comparing CSP with conventional BiVP in patients with HFrEF undergoing CRT. Four main findings emerged: (i) CSP resulted in a 49% lower risk of the composite endpoint of HHF or all-cause mortality compared with BiVP (OR 0.51, 95% CI 0.28–0.93), corresponding to 69 fewer composite events per 1000 patients treated; (ii) no significant differences were observed for HHF alone or all-cause mortality when the component outcomes were analysed separately; (iii) CSP was associated with greater improvement in functional capacity, reflected by longer 6-min walk distance and better NYHA functional class, although QoL measures did not differ significantly; and (iv) CSP achieved greater QRS-duration reduction, whereas echocardiographic remodelling indices, pacing threshold, procedural metrics, complications, and reintervention were broadly comparable between strategies.

The results should be interpreted in the context of the extensive evidence base supporting conventional BiVP. Landmark CRT trials established that electrical resynchronization improves symptoms, reverses adverse LV remodelling, reduces HHF, and, in selected populations, improves survival in patients with HFrEF and electrical dyssynchrony.^1–6,39–42^ Therefore, the present findings do not undermine the established role of BiVP as the reference standard for CRT; rather, they address whether more physiological recruitment of the His–Purkinje system may further optimize resynchronization in appropriately selected patients. BiVP achieves resynchronization indirectly through epicardial LV activation via the coronary venous system, a strategy that may be constrained by venous anatomy, LV lead position, phrenic nerve capture, myocardial scar, and residual conduction delay.^43,44^ Conduction system pacing, particularly when true His–Purkinje capture is achieved, aims to correct conduction delay within the native conduction system and may therefore provide more physiological ventricular activation.^45,46^

This mechanistic distinction provides a plausible explanation for the greater QRS narrowing and functional improvement observed with CSP in the present analysis. However, the absence of consistent superiority in LVEF, LVESV, and QoL underscores that electrical resynchronization may not uniformly translate into structural remodelling or patient-reported benefit across heterogeneous HFrEF populations. Cardiac resynchronization therapy response is influenced by multiple interacting factors, including conduction substrate, scar burden, LV geometry, atrioventricular timing, mitral valve mechanics, atrial rhythm, right ventricular function, and device optimization.^47–52^ In addition, echocardiographic remodelling endpoints are sensitive to imaging protocols, loading conditions, measurement variability, and follow-up duration.^53^ In patients with extensive fibrosis or ischaemic scar, improved activation may enhance electromechanical efficiency without necessarily producing large changes in global chamber volumes, because scar burden and scar location remain important determinants of CRT response.^54–56^ Thus, functional improvement may precede, or occur without, clear superiority in conventional remodelling indices, particularly in modest-sized trials with limited follow-up.^57,58^

The clinical outcome findings require similarly cautious interpretation. Although CSP was associated with a lower composite risk of HHF or all-cause mortality, neither HHF nor mortality differed significantly when assessed as individual endpoints. This pattern likely reflects limited event accrual, the relatively modest sample size of the available randomized evidence, and the short-to-intermediate follow-up of most trials. The composite endpoint may be more sensitive to early differences in HF progression, whereas mortality effects generally require larger populations and longer observation. Accordingly, the observed composite benefit should be regarded as supportive but not definitive evidence of clinical advantage. The most appropriate interpretation is that CSP may attenuate clinically relevant HF deterioration in selected patients, while its effect on survival remains uncertain.

The heterogeneity of the randomized evidence also deserves emphasis. Recent trials have yielded apparently divergent results. HeartSync-LBBP reported favourable long-term outcomes with LBBP in patients with LBBB and severely reduced LVEF,^13^ whereas PhysioSync-HF did not support routine first-line CSP in HFrEF with LBBB,^31^ and LEFT-BUNDLE-CRT did not demonstrate non-inferiority of LBBAP-CRT compared with BiVP-CRT in the intention-to-treat analysis despite high response rates in both groups.^15^ These findings are not necessarily contradictory. Differences in patient phenotype, ischaemic vs. nonischaemic substrate, CSP modality, capture validation, crossover, operator experience, endpoint definition, follow-up duration, and background guideline-directed medical therapy (GDMT) may all influence the apparent treatment effect. Therefore, the present meta-analysis supports CSP as a promising resynchronization strategy, but not as a uniform replacement for BiVP across all CRT-eligible patients.

These findings should also be interpreted in light of procedural and implementation-related considerations. The absence of significant differences in complications, reintervention, pacing thresholds, procedure time, and fluoroscopy time is reassuring, but should not be interpreted as proof of long-term equivalence. His bundle pacing remains susceptible to threshold elevation, sensing limitations, lead revision, and implications for battery longevity.^59,60^ Left bundle branch area pacing generally provides more favourable acute and mid-term electrical parameters, but introduces distinct considerations related to septal lead depth, acute or delayed septal perforation, potential LV-cavity exposure, thromboembolic risk in the event of perforation, late threshold behaviour, long-term lead integrity, and extraction safety.^61–63^ Device platform may also influence outcomes, as evidence derived from lumenless leads may not be directly generalizable to stylet-driven lead systems.^64,65^

The reproducibility of LBBP/LBBAP outcomes in routine practice is also likely to depend on procedural experience and rigorous capture adjudication. Reported LBBP implantation success varies substantially across studies, with higher success rates generally reported in experienced centers.^13,62,63,66,67^ Contemporary real-world data also support the feasibility and clinical relevance of LBBAP outside highly selected trial settings. In the TREEBEARD registry-based trial, Bertini et al. reported that LBBAP was associated with a lower 2-year composite risk of cardiovascular death or heart failure hospitalization compared with traditional right ventricular pacing in middle-aged adults, with similar procedural safety.^68^ Although this study did not directly compare CSP with BiVP for CRT in HFrEF, it supports the feasibility and clinical relevance of LBBAP in contemporary practice. At the same time, marked variability in procedural adoption and volume across regions and centres, as highlighted by the ESC-EHRA Atlas, reinforces the importance of local expertise and institutional experience.^69^ Learning-curve analyses further suggest that LBBAP proficiency requires substantial cumulative procedural exposure,^63,70^ while the updated EHRA core curriculum emphasizes the relevance of structured training for contemporary device therapy, including CSP.^71^

A further consideration is that CSP is not a uniform intervention. It encompasses HBP, LBBP, LBBAP, LV septal pacing, and, in some studies, deep septal pacing without confirmed conduction system capture. The EHRA implantation consensus emphasizes that confirmation of capture requires integration of lead position, paced QRS morphology, potential-to-QRS timing, output-dependent transitions, and 12-lead ECG assessment.^17^ Anatomical septal lead placement alone is insufficient to establish His–Purkinje recruitment. This issue is particularly relevant in patients with structural heart disease, in whom LBBP implementation should be individualized according to conduction substrate, septal anatomy, myocardial disease, and the ability to document true conduction system capture. Recent expert discussion has emphasized the distinction between anatomic deep septal pacing and functional conduction system recruitment, as well as the need for rigorous intraprocedural and post-implant assessment.^72^ Thus, the clinical value of CSP depends not only on the theoretical advantages of conduction-system recruitment but also on operator training and expertise, capture adjudication, substrate selection, device platform, and institutional procedural maturity.

Taken together, these data support a cautious but clinically relevant interpretation. Conventional BiVP remains the reference standard, supported by the largest body of randomized evidence and extensive clinical experience. Conduction system pacing should not yet be viewed as a routine replacement for BiVP in all eligible patients, but it should also no longer be regarded merely as an experimental or bailout strategy. In patients with typical LBBB, limited scar burden, nonischaemic cardiomyopathy, a correctable conduction abnormality, and implantation by experienced operators able to confirm true conduction system capture, CSP may provide clinically meaningful benefit. Conversely, in patients with diffuse myocardial disease, non-LBBB intraventricular conduction delay, substantial scar, uncertain or unstable capture, or treatment in centres early in the CSP learning curve, BiVP may remain the more reliable resynchronization strategy. Adequately powered randomized trials with standardized capture criteria, harmonized endpoint definitions, longer follow-up, and clinically meaningful outcomes are needed to define the role of CSP in contemporary CRT practice.

### Limitations

Several limitations should be acknowledged. First, this was an aggregate trial-level meta-analysis and therefore could not examine patient-level effect modifiers, including LBBB morphology, QRS duration, ischaemic vs. nonischaemic aetiology, scar burden, sex, atrial rhythm, PR interval, LV size, or background GDMT. Second, the randomized evidence base remains modest, with limited event accrual for HHF and all-cause mortality. Consequently, all certainty ratings were downgraded for imprecision, and conclusions regarding hard clinical outcomes should be interpreted with appropriate caution. Third, CSP represented a heterogeneous intervention across trials, encompassing HBP, LBBP/LBBAP, and mixed CSP approaches, with variable criteria for confirming true conduction system capture. This distinction is clinically important because septal lead position does not necessarily equate to true conduction system capture. Fourth, some included trials enrolled patients undergoing AV node ablation, representing an additional source of clinical heterogeneity because these patients may differ from the typical HFrEF-CRT population with respect to rhythm status, clinical outcomes, baseline QRS duration, and QRS morphology. However, these studies fulfilled the prespecified eligibility criteria, and because the analysis pooled randomized within-trial comparisons, randomization should have minimized baseline QRS-related imbalance between the CSP and BiVP groups within each trial. Fifth, endpoint definitions and methods of outcome assessment varied across the included trials, particularly for clinical, functional, QoL, imaging, procedural, and safety outcomes, potentially attenuating between-study comparability and influencing the pooled estimates. Sixth, follow-up duration differed substantially among studies and was generally short to intermediate, which may have affected the estimation of time-dependent outcomes and limited inference regarding long-term lead performance, battery longevity, late threshold behaviour, extraction safety, and infrequent but clinically important complications. Seventh, procedural outcomes may have been influenced by operator experience, centre volume, and institutional learning curves, which were not consistently reported across trials. Finally, QoL instruments and imaging protocols varied among studies, reducing power to detect patient-centred and remodelling differences.

## Conclusions

Among patients with HFrEF undergoing CRT, moderate-certainty evidence indicates that CSP results in a lower composite risk of HHF or all-cause mortality compared with BiVP. Conduction system pacing is also associated with improved functional capacity, without apparent differences in safety or procedural outcomes between strategies. Larger-scale, adequately powered randomized trials are needed to establish the effect of CSP on hard clinical endpoints with greater certainty.

## Supplementary Material

euag170_Supplementary_Data

## Data Availability

The data underlying this article will be shared on reasonable request to the corresponding author. The items outlined in the PRISMA 2020 checklist and their corresponding locations within the manuscript are presented in Supplementary material online, *Table S7*.

## Protocol

The study protocol was prospectively registered in OSF (https://osf.io/k3j2u)
