# Supplementary material for: Conduction system vs. biventricular pacing in heart failure with reduced ejection fraction: a meta-analysis of randomized controlled trials
Source: Europace. 2026 Aug 13;28(8):euag170. doi: 10.1093/europace/euag170 (PMC13466950; doi:10.1093/europace/euag170)
Supplement: Supplementary file 1 [file euag170_supplementary_data.docx]

**Supplementary material**

**Conduction System Versus Biventricular Pacing in Heart Failure With Reduced Ejection Fraction: A Meta-Analysis of Randomized Controlled Trials**

**Contents**

[Supplemental Table 1. Search strategy for MEDLINE (Pubmed)	4](#_Toc228555537)

[Supplemental Table 2. Search strategy for Embase	4](#_Toc228555538)

[Supplemental Table 3. Search strategy for Cochrane library	5](#_Toc228555539)

[Supplemental Table 4. Search strategy for Scopus	6](#_Toc228555540)

[Supplementary Table 5. Study-level and participant characteristics.	7](#_Toc228555541)

[Supplemental Figure 1. PRISMA 2020 flow diagram of study selection.	8](#_Toc228555542)

[Supplemental Figure 2. Risk-of-bias assessment of included randomized controlled trials using the RoB 2 tool.	9](#_Toc228555543)

[Supplemental Figure 3. Forest plots showing the comparative effect of conduction system pacing (CSP) versus biventricular pacing (BiVP) on (A) any complication and (B) reintervention.	10](#_Toc228555544)

[Supplemental Figure 4. Forest plot showing the comparative effect of conduction system pacing (CSP) versus biventricular pacing (BiVP) on total procedure time.	11](#_Toc228555545)

[Supplemental Figure 5. Forest plot showing the comparative effect of conduction system pacing (CSP) versus biventricular pacing (BiVP) on fluoroscopy time.	11](#_Toc228555546)

[Supplemental Figure 6. Baujat plot for the composite endpoint of HHF or all-cause mortality.	12](#_Toc228555547)

[Supplemental Figure 7. Influence diagnostic plot for the composite endpoint of HHF or all-cause mortality.	12](#_Toc228555548)

[Supplemental Figure 8. Leave-one-out analysis for the composite endpoint of HHF or all-cause mortality.	13](#_Toc228555549)

[Supplemental Figure 9. Baujat plot for HHF.	13](#_Toc228555550)

[Supplemental Figure 10. Influence diagnostic plot for HHF.	14](#_Toc228555551)

[Supplemental Figure 11. Leave-one-out analysis for HHF.	14](#_Toc228555552)

[Supplemental Figure 12. Baujat plot for all-cause mortality.	15](#_Toc228555553)

[Supplemental Figure 13. Influence diagnostic plot for all-cause mortality.	15](#_Toc228555554)

[Supplemental Figure 14. Leave-one-out analysis for all-cause mortality.	16](#_Toc228555555)

[Supplemental Figure 15. Baujat plot for 6-minute walk distance.	16](#_Toc228555556)

[Supplemental Figure 16. Influence diagnostic plot for 6-minute walk distance.	17](#_Toc228555557)

[Supplemental Figure 17. Leave-one-out analysis for 6-minute walk distance.	17](#_Toc228555558)

[Supplemental Figure 18. Baujat plot for NYHA class.	18](#_Toc228555559)

[Supplemental Figure 19. Influence diagnostic plot for NYHA class.	18](#_Toc228555560)

[Supplemental Figure 20. Leave-one-out analysis for NYHA class.	19](#_Toc228555561)

[Supplemental Figure 21. Baujat plot for quality of life.	19](#_Toc228555562)

[Supplemental Figure 22. Influence diagnostic plot for quality of life.	20](#_Toc228555563)

[Supplemental Figure 23. Leave-one-out analysis for quality of life.	20](#_Toc228555564)

[Supplemental Figure 24. Baujat plot for QRS duration.	21](#_Toc228555565)

[Supplemental Figure 25. Influence diagnostic plot for QRS duration	21](#_Toc228555566)

[Supplemental Figure 26. Leave-one-out analysis for QRS duration.	22](#_Toc228555567)

[Supplemental Figure 27. Baujat plot for left ventricular ejection fraction.	22](#_Toc228555568)

[Supplemental Figure 28. Influence diagnostic plot for left ventricular ejection fraction	23](#_Toc228555569)

[Supplemental Figure 29. Leave-one-out analysis for left ventricular ejection fraction.	23](#_Toc228555570)

[Supplemental Figure 30. Baujat plot for left ventricular end-systolic volume.	24](#_Toc228555571)

[Supplemental Figure 31. Influence diagnostic plot for left ventricular end-systolic volume.	24](#_Toc228555572)

[Supplemental Figure 32. Leave-one-out analysis for left ventricular end-systolic volume.	25](#_Toc228555573)

[Supplemental Figure 33. Baujat plot for pacing threshold.	25](#_Toc228555574)

[Supplemental Figure 34. Influence diagnostic plot for pacing threshold.	26](#_Toc228555575)

[Supplemental Figure 35. Leave-one-out analysis for pacing threshold.	26](#_Toc228555576)

[Supplemental Figure 36. Baujat plot for any complication.	27](#_Toc228555577)

[Supplemental Figure 37. Influence diagnostic plot for any complication.	27](#_Toc228555578)

[Supplemental Figure 38. Leave-one-out analysis for any complication.	28](#_Toc228555579)

[Supplemental Figure 39. Baujat plot for reintervention.	28](#_Toc228555580)

[Supplemental Figure 40. Influence diagnostic plot for reintervention.	29](#_Toc228555581)

[Supplemental Figure 41. Leave-one-out analysis for reintervention.	29](#_Toc228555582)

[Supplemental Figure 42. Baujat plot for total procedure time.	30](#_Toc228555583)

[Supplemental Figure 43. Influence diagnostic plot for total procedure time.	30](#_Toc228555584)

[Supplemental Figure 44. Leave-one-out analysis for total procedure time.	31](#_Toc228555585)

[Supplemental Figure 45. Baujat plot for fluoroscopy time.	31](#_Toc228555586)

[Supplemental Figure 46. Influence diagnostic plot for fluoroscopy time.	32](#_Toc228555587)

[Supplemental Figure 47. Leave-one-out analysis for fluoroscopy time.	32](#_Toc228555588)

[Supplemental Figure 48. Contour-enhanced funnel plot assessing small-study effects for the comparison of conduction system pacing versus biventricular pacing on HHF. Individual studies are plotted according to risk ratio and standard error, with shaded regions denoting conventional levels of statistical significance. The vertical dashed line represents the pooled effect estimate, and diagonal dashed lines indicate the pseudo-95% confidence limits.	33](#_Toc228555589)

[Supplemental Figure 49. Contour-enhanced funnel plot assessing small-study effects for the comparison of conduction system pacing versus biventricular pacing on all-cause mortality.	34](#_Toc228555590)

[Supplemental Figure 50. Contour-enhanced funnel plot assessing small-study effects for the comparison of conduction system pacing versus biventricular pacing on QRS duration.	35](#_Toc228555591)

[Supplemental Figure 51. Contour-enhanced funnel plot assessing small-study effects for the comparison of conduction system pacing versus biventricular pacing on left ventricular ejection fraction.	36](#_Toc228555592)

[Supplemental Figure 52. Contour-enhanced funnel plot assessing small-study effects for the comparison of conduction system pacing versus biventricular pacing on pacing threshold.	37](#_Toc228555593)

[Supplemental Figure 53. Contour-enhanced funnel plot assessing small-study effects for the comparison of conduction system pacing versus biventricular pacing on total procedure time.	38](#_Toc228555594)

[Supplemental Figure 54. Contour-enhanced funnel plot assessing small-study effects for the comparison of conduction system pacing versus biventricular pacing on any complication.	39](#_Toc228555595)

[Supplemental Table 6. Evaluation of certainty of evidence with GRADEpro GDT for CSP compared to BiVP in HFrEF.	40](#_Toc228555596)

[Supplemental Table 7. PRISMA 2020 Checklist	42](#_Toc228555597)

# **Supplemental Table 1.** Search strategy for MEDLINE (Pubmed)

("conduction-system pacing"[Title/Abstract] OR "conduction-system pacing"[Title/Abstract] OR "CSP"[Title/Abstract] OR "his bundle pacing"[Title/Abstract] OR "his bundle pacing"[Title/Abstract] OR "HBP"[Title/Abstract] OR "left bundle-branch pacing"[Title/Abstract] OR "left bundle-branch pacing"[Title/Abstract] OR "LBBP"[Title/Abstract] OR "left bundle-branch area pacing"[Title/Abstract] OR "left bundle-branch area pacing"[Title/Abstract] OR "LBBAP"[Title/Abstract] OR "LBAP"[Title/Abstract] OR "left ventricular septal pacing"[Title/Abstract] OR "LVSP"[Title/Abstract] OR "deep septal pacing"[Title/Abstract] OR "DSP"[Title/Abstract] OR "HOT-CRT"[Title/Abstract] OR "LOT-CRT"[Title/Abstract]) AND ("biventricular pacing"[Title/Abstract] OR "bi-ventricular pacing"[Title/Abstract] OR "biventricular cardiac pacing"[Title/Abstract] OR "BiVP"[Title/Abstract] OR "BiV pacing"[Title/Abstract] OR "Cardiac Resynchronization Therapy"[Title/Abstract] OR "Cardiac Resynchronization Therapy"[MeSH Terms] OR "CRT"[Title/Abstract] OR "CRT-P"[Title/Abstract] OR "CRT-D"[Title/Abstract]) AND ("Heart Failure"[Title/Abstract] OR "Heart Failure"[MeSH Terms] OR "HFrEF"[Title/Abstract] OR "heart failure with reduced ejection fraction"[Title/Abstract] OR "reduced ejection fraction"[Title/Abstract] OR "left ventricular systolic dysfunction"[Title/Abstract] OR "ventricular dysfunction, left"[MeSH Terms] OR "cardiomyopathy"[Title/Abstract]) AND ("randomized controlled trial"[Publication Type] OR "controlled clinical trial"[Publication Type] OR "random*"[Title/Abstract] OR "randomised"[Title/Abstract] OR "randomized"[Title/Abstract] OR "randomly"[Title/Abstract] OR "trial"[Title/Abstract] OR "clinical trial"[Publication Type])

# **Supplemental Table 2.** Search strategy for Embase

( 'conduction system pacing':ti,ab,kw

OR 'conduction-system pacing':ti,ab,kw

OR csp:ti,ab,kw

OR 'his bundle pacing':ti,ab,kw

OR 'his-bundle pacing':ti,ab,kw

OR hbp:ti,ab,kw

OR 'left bundle branch pacing':ti,ab,kw

OR 'left bundle-branch pacing':ti,ab,kw

OR lbbp:ti,ab,kw

OR 'left bundle branch area pacing':ti,ab,kw

OR 'left bundle-branch area pacing':ti,ab,kw

OR lbbap:ti,ab,kw

OR lbap:ti,ab,kw

OR 'left ventricular septal pacing':ti,ab,kw

OR lvsp:ti,ab,kw

OR 'deep septal pacing':ti,ab,kw

OR dsp:ti,ab,kw

OR 'his optimized cardiac resynchronization therapy':ti,ab,kw

OR 'his-optimized cardiac resynchronization therapy':ti,ab,kw

OR 'hot-crt':ti,ab,kw

OR 'left bundle optimized cardiac resynchronization therapy':ti,ab,kw

OR 'left bundle-optimized cardiac resynchronization therapy':ti,ab,kw

OR 'lot-crt':ti,ab,kw)

AND

( 'biventricular pacing':ti,ab,kw

OR 'bi-ventricular pacing':ti,ab,kw

OR 'biventricular cardiac pacing':ti,ab,kw

OR bivp:ti,ab,kw

OR 'biv pacing':ti,ab,kw

OR 'cardiac resynchronization therapy'/exp

OR 'cardiac resynchronization therapy':ti,ab,kw

OR crt:ti,ab,kw

OR 'crt-p':ti,ab,kw

OR 'crt-d':ti,ab,kw)

AND

( 'heart failure'/exp

OR 'heart failure':ti,ab,kw

OR hfref:ti,ab,kw

OR 'heart failure with reduced ejection fraction':ti,ab,kw

OR 'reduced ejection fraction':ti,ab,kw

OR 'left ventricular systolic dysfunction':ti,ab,kw

OR 'left ventricle dysfunction'/exp

OR cardiomyopathy:ti,ab,kw)

AND

( 'randomized controlled trial'/exp

OR 'controlled clinical trial'/exp

OR 'clinical trial'/exp

OR random*:ti,ab,kw

OR randomised:ti,ab,kw

OR randomized:ti,ab,kw

OR randomly:ti,ab,kw

OR trial:ti,ab,kw)

# **Supplemental Table 3.** Search strategy for Cochrane library

#1 MeSH descriptor: [Cardiac Resynchronization Therapy] explode all trees

#2

(

"biventricular pacing"

OR "bi-ventricular pacing"

OR "biventricular cardiac pacing"

OR BiVP

OR "BiV pacing"

OR "cardiac resynchronization therapy"

OR "cardiac resynchronisation therapy"

OR CRT

OR "CRT-P"

OR "CRT-D"

):ti,ab,kw

#3 #1 OR #2

#4

(

"conduction system pacing"

OR "conduction-system pacing"

OR CSP

OR "His bundle pacing"

OR "His-bundle pacing"

OR HBP

OR "left bundle branch pacing"

OR "left bundle-branch pacing"

OR LBBP

OR "left bundle branch area pacing"

OR "left bundle-branch area pacing"

OR LBBAP

OR LBAP

OR "left ventricular septal pacing"

OR LVSP

OR "deep septal pacing"

OR DSP

OR "His optimized cardiac resynchronization therapy"

OR "His-optimized cardiac resynchronization therapy"

OR "His optimised cardiac resynchronisation therapy"

OR "HOT-CRT"

OR "left bundle optimized cardiac resynchronization therapy"

OR "left bundle-optimized cardiac resynchronization therapy"

OR "left bundle optimised cardiac resynchronisation therapy"

OR "LOT-CRT"

):ti,ab,kw

#5 MeSH descriptor: [Heart Failure] explode all trees

#6 MeSH descriptor: [Ventricular Dysfunction, Left] explode all trees

#7

(

"heart failure"

OR HFrEF

OR "heart failure with reduced ejection fraction"

OR "reduced ejection fraction"

OR "left ventricular systolic dysfunction"

OR cardiomyopathy

):ti,ab,kw

#8 #5 OR #6 OR #7

#9 #3 AND #4 AND #8

# **Supplemental Table 4.** Search strategy for Scopus

TITLE-ABS-KEY ( ( "conduction system pacing" OR "His bundle pacing" OR "His-bundle pacing" OR HBP OR "left bundle branch pacing" OR LBBP OR "left bundle branch area pacing" OR LBBAP OR LBAP OR "left ventricular septal pacing" OR LVSP OR "deep septal pacing" OR DSP OR "HOT-CRT" OR "LOT-CRT" ) AND ( "biventricular pacing" OR "bi-ventricular pacing" OR BiVP OR "cardiac resynchronization therapy" OR "cardiac resynchronisation therapy" OR CRT ) AND ( "heart failure" OR HFrEF OR "heart failure with reduced ejection fraction" OR "left ventricular systolic dysfunction" OR cardiomyopathy ) AND ( random* OR randomized OR randomised OR trial OR crossover OR "cross-over" ) )

# **Supplementary Table 5.** Study-level and participant characteristics.

| **First author (study)-year** | **Study type** | **Population** | **Total (n)** | **AF, % (I/C)** | **BMI, kg/m² mean (SD) (I/C)** | **β-blockers, % (I/C)** |
| --- | --- | --- | --- | --- | --- | --- |
| Lustgarten DL-2015 | RCT (crossover) | Adults (≥18 years) with QRS>130 ms and CRT indication | 29 | 0 | 29.5 (9.3) | NR |
| Upadhyay GA (His-SYNC)-2019 | RCT | Adults with HF and CRT I/IIa indication per ACCF/AHA/HRS | 40 | 25 / 38 | NR | 94 / 100 |
| Vinther M (His-Alternative)-2021 | RCT | Patients with HF (LVEF≤35%, NYHA II-IV) and Strauss LBBB | 50 | NR | 26.1 (4.4) / 26.5 (4.6) | 92 / 80 |
| Pujol-Lopez M (LEVEL-AT)-2022 | RCT | Adults with HF (LVEF≤35%, NYHA II-IV) and Strauss LBBB QRS≥130 ms or non-LBBB QRS≥150 ms or AVB plus cardiac dysfunction | 70 | 7 / 8 | 26.9 (4) / 28.3 (4) | 83 / 89 |
| Wang Y (LBBB-RESYNC)-2022 | RCT | Adults (18-80 years) in SR with nonischemic HF (NYHA II-IV) and Strauss LBBB | 40 | NR | NR | 95 |
| Huang W (ALTERNATIVE-AF)-2022 | RCT (crossover) | Patients with HF (LVEF≤40%, NYHA II-IV) and persistent AF with QRS<120 ms | 50 (25 in each group) | 100 | NR | NR |
| Vijayaraman P (HOT-CRT)-2023 | RCT | Adults (>18 years) with HF and: i) LVEF ≤35% with LBBB (QRS duration>120 ms) or non-LBBB (QRS >150 ms) or ii) LVEF ≤50% with >40% RV pacing. | 100 | NR | NR | 90 / 94 |
| Pujol-Lopez M (CONSYST-CRT)-2025 | RCT | Adults with HF (LVEF≤35%, NYHA II-IV) and Strauss LBBB QRS≥130 ms or non-LBBB QRS≥150 ms or AVB plus cardiac dysfunction (LVEF<40%) | 134 | 22 / 18 | NR | NR |
| Zizek D (CSP-SYNC)-2025 | RCT | Patients with HF (LVEF≤35%, NYHA II-IV) and class I CRT indication due to Strauss LBBB | 62 | NR | 28.4 (5.4) / 28.4 (7.8) | 100 / 97 |
| Cano O (LEFT-BUNDLE CRT)-2026 | RCT | Adults (>18 years) with LBBB and class I/IIa CRT indication per ESC or ACC/AHA/HRS | 175 | NR | 28.4 (4.3) / 27.8 (4.8) | 86 |
| Zimerman A (PhysioSync-HF)-2026 | RCT | Adults (>18 years) with HF (LVEF≤35%, NYHA II-III) and LBBB (QRS≥130 ms in women and ≥140 ms in men) on OMT | 173 | 8 / 6 | NR | 91 / 88 |
| Chen X (HeartSync-LBBP)-2026 | RCT | Patients with HF (LVEF≤35%, NYHA II-IV) and LBBB | 200 | NR | NR | 96 / 95 |
| le Polain de Waroux JB (LECART)-2026 | RCT | Patients with HF (LVEF≤40%, NYHA II-IV) and non-RBBB with CRT indication (ESC Guidelines) | 168 | NR | NR | NR |

Abbreviations: ACC, American College of Cardiology; ACCF, American College of Cardiology Foundation; AF, atrial fibrillation; AHA, American Heart Association; AVB, atrioventricular block; BMI, body mass index; CRT, cardiac resynchronization therapy; ESC, European Society of Cardiology; HF, heart failure; HRS, Heart Rhythm Society; I/C, intervention/control; LBBB, left bundle branch block; LVEF, left ventricular ejection fraction; NR, not reported; NYHA, New York Heart Association; OMT, optimal medical therapy; RBBB, right bundle branch block; RCT, randomized controlled trial; RV, right ventricular; SD, standard deviation; SR, sinus rhythm.

# **Supplemental Figure 1**. PRISMA 2020 flow diagram of study selection.

**Supplemental Figure 2**. Risk-of-bias assessment of included randomized controlled trials using the RoB 2 tool.

Risk of bias was evaluated across five domains: D1, bias arising from the randomization process; D2, bias due to deviations from intended interventions; D3, bias due to missing outcome data; D4, bias in measurement of the outcome; and D5, bias in selection of the reported result. Green circles with a plus sign indicate low risk of bias, whereas yellow circles with a minus sign indicate some concerns.

**Supplemental Figure 3**. Forest plots showing the comparative effect of conduction system pacing (CSP) versus biventricular pacing (BiVP) on (A) any complication and (B) reintervention. Effect estimates are presented as odds ratios (ORs) with 95% confidence intervals (CIs), pooled using random-effects models and stratified by CSP approach, including His bundle pacing (HBP) and left bundle branch area pacing (LBBAP). Squares represent study-specific estimates, with size proportional to study weight; horizontal lines indicate 95% CIs; diamonds indicate pooled estimates. Values <1 favor CSP, whereas values >1 favor BiVP.

**Supplemental Figure 4**. Forest plot showing the comparative effect of conduction system pacing (CSP) versus biventricular pacing (BiVP) on total procedure time. Effect estimates are presented as mean differences (MDs) with 95% confidence intervals (CIs), pooled using random-effects models and stratified by CSP approach, including His bundle pacing (HBP) and left bundle branch area pacing (LBBAP).

**Supplemental Figure 5**. Forest plot showing the comparative effect of conduction system pacing (CSP) versus biventricular pacing (BiVP) on fluoroscopy time. Effect estimates are presented as mean differences (MDs) with 95% confidence intervals (CIs), pooled using random-effects models and stratified by CSP approach, including His bundle pacing (HBP) and left bundle branch area pacing (LBBAP). Squares represent study-specific estimates, with size proportional to study weight; horizontal lines indicate 95% CIs; diamonds indicate pooled estimates. Negative values favor CSP, whereas positive values favor BiVP.

# **Supplemental Figure 6**. Baujat plot for the composite endpoint of HHF or all-cause mortality.

# **Supplemental Figure 7**. Influence diagnostic plot for the composite endpoint of HHF or all-cause mortality.

# **Supplemental Figure 8**. Leave-one-out analysis for the composite endpoint of HHF or all-cause mortality.

# **Supplemental Figure 9**. Baujat plot for HHF.

# **Supplemental Figure 10**. Influence diagnostic plot for HHF.

# **Supplemental Figure 11**. Leave-one-out analysis for HHF.

# **Supplemental Figure 12**. Baujat plot for all-cause mortality.

# **Supplemental Figure 13**. Influence diagnostic plot for all-cause mortality.

# **Supplemental Figure 14**. Leave-one-out analysis for all-cause mortality.

# **Supplemental Figure 15**. Baujat plot for 6-minute walk distance.

# **Supplemental Figure 16**. Influence diagnostic plot for 6-minute walk distance.

# **Supplemental Figure 17**. Leave-one-out analysis for 6-minute walk distance.

# **Supplemental Figure 18**. Baujat plot for NYHA class.

# **Supplemental Figure 19**. Influence diagnostic plot for NYHA class.

# **Supplemental Figure 20**. Leave-one-out analysis for NYHA class.

# **Supplemental Figure 21**. Baujat plot for quality of life.

# **Supplemental Figure 22**. Influence diagnostic plot for quality of life.

# **Supplemental Figure 23**. Leave-one-out analysis for quality of life.

# **Supplemental Figure 24**. Baujat plot for QRS duration.

**Supplemental Figure 25**. Influence diagnostic plot for QRS duration.

# **Supplemental Figure 26**. Leave-one-out analysis for QRS duration.

# **Supplemental Figure 27**. Baujat plot for left ventricular ejection fraction.

**Supplemental Figure 28**. Influence diagnostic plot for left ventricular ejection fraction.

# **Supplemental Figure 29**. Leave-one-out analysis for left ventricular ejection fraction.

# **Supplemental Figure 30**. Baujat plot for left ventricular end-systolic volume.

# **Supplemental Figure 31**. Influence diagnostic plot for left ventricular end-systolic volume.

# **Supplemental Figure 32**. Leave-one-out analysis for left ventricular end-systolic volume.

# **Supplemental Figure 33**. Baujat plot for pacing threshold.

# **Supplemental Figure 34**. Influence diagnostic plot for pacing threshold.

# **Supplemental Figure 35**. Leave-one-out analysis for pacing threshold.

# **Supplemental Figure 36**. Baujat plot for any complication.

# **Supplemental Figure 37**. Influence diagnostic plot for any complication.

# **Supplemental Figure 38**. Leave-one-out analysis for any complication.

# **Supplemental Figure 39**. Baujat plot for reintervention.

# **Supplemental Figure 40**. Influence diagnostic plot for reintervention.

# **Supplemental Figure 41**. Leave-one-out analysis for reintervention.

# **Supplemental Figure 42**. Baujat plot for total procedure time.

# **Supplemental Figure 43**. Influence diagnostic plot for total procedure time.

# **Supplemental Figure 44**. Leave-one-out analysis for total procedure time.

# **Supplemental Figure 45**. Baujat plot for fluoroscopy time.

# **Supplemental Figure 46**. Influence diagnostic plot for fluoroscopy time.

# **Supplemental Figure 47**. Leave-one-out analysis for fluoroscopy time.

# **Supplemental Figure 48**. Contour-enhanced funnel plot assessing small-study effects for the comparison of conduction system pacing versus biventricular pacing on HHF. Individual studies are plotted according to odds ratio and standard error, with shaded regions denoting conventional levels of statistical significance. The vertical dashed line represents the pooled effect estimate, and diagonal dashed lines indicate the pseudo-95% confidence limits.

# **Supplemental Figure 49**. Contour-enhanced funnel plot assessing small-study effects for the comparison of conduction system pacing versus biventricular pacing on all-cause mortality.

# **Supplemental Figure 50**. Contour-enhanced funnel plot assessing small-study effects for the comparison of conduction system pacing versus biventricular pacing on QRS duration.

# **Supplemental Figure 51**. Contour-enhanced funnel plot assessing small-study effects for the comparison of conduction system pacing versus biventricular pacing on left ventricular ejection fraction.

# **Supplemental Figure 52**. Contour-enhanced funnel plot assessing small-study effects for the comparison of conduction system pacing versus biventricular pacing on pacing threshold.

# **Supplemental Figure 53**. Contour-enhanced funnel plot assessing small-study effects for the comparison of conduction system pacing versus biventricular pacing on total procedure time.

# **Supplemental Figure 54**. Contour-enhanced funnel plot assessing small-study effects for the comparison of conduction system pacing versus biventricular pacing on any complication.

# **Supplemental Table 6**. Evaluation of certainty of evidence with GRADEpro GDT for CSP compared to BiVP in HFrEF.

| **Certainty assessment** | | | | | | | **Summary of findings** | | | | |
| --- | --- | --- | --- | --- | --- | --- | --- | --- | --- | --- | --- |
| **Participants (studies) Follow-up** | **Risk of bias** | **Inconsistency** | **Indirectness** | **Imprecision** | **Publication bias** | **Overall certainty of evidence** | **Study event rates (%)** | | **Relative effect (95% CI)** | **Anticipated absolute effects** | |
|  |  |  |  |  |  |  | **With BiVP** | **With CSP** |  | **Risk with BiVP** | **Risk difference with CSP** |
| **Composite of HHF or all-cause mortality** | | | | | | | | | | | |
| 1071 (9 RCTs) | not serious | not serious | not serious | serious^a^ | none | ⨁⨁⨁◯ Moderate^a^ | 83/538 (15.4%) | 46/533 (8.6%) | **OR 0.51** (0.28 to 0.93) | 83/538 (15.4%) | **69 fewer per 1.000** (from 106 fewer to 9 fewer) |
| **HHF** | | | | | | | | | | | |
| 1221 (11 RCTs) | not serious | not serious | not serious | serious^b^ | none | ⨁⨁⨁◯ Moderate^b^ | 69/613 (11.3%) | 43/608 (7.1%) | **OR 0.68** (0.36 to 1.27) | 69/613 (11.3%) | **33 fewer per 1.000** (from 69 fewer to 26 more) |
| **All-cause mortality** | | | | | | | | | | | |
| 1261 (12 RCTs) | not serious | not serious | not serious | serious^c^ | none | ⨁⨁⨁◯ Moderate^c^ | 32/637 (5.0%) | 24/624 (3.8%) | **OR 0.75** (0.36 to 1.58) | 32/637 (5.0%) | **12 fewer per 1.000** (from 32 fewer to 27 more) |
| **6-minute walk distance** | | | | | | | | | | | |
| 649 (7 RCTs) | not serious | not serious | not serious | serious^d^ | none | ⨁⨁⨁◯ Moderate^d^ | -/307 | -/342 | **MD 21.53** (4.33 to 38.73) | -/307 |  |
| **NYHA class** | | | | | | | | | | | |
| 582 (7 RCTs) | not serious | not serious | not serious | serious^e^ | none | ⨁⨁⨁◯ Moderate^e^ | -/292 | -/290 | **SMD −0.27** (−0.47 to −0.07) | -/292 |  |
| **Quality of life** | | | | | | | | | | | |
| 578 (6 RCTs) | not serious | not serious | not serious | serious^f^ | none | ⨁⨁⨁◯ Moderate^f^ | -/286 | -/292 | **SMD −0.06** (−0.22 to 0.11) | -/286 |  |

**CI:** confidence interval; **OR:** odds ratio; **MD**: mean difference; **SMD**: standardized mean difference.

#### Explanations

a. Downgraded one level for imprecision because the 95% CI crossed the prespecified threshold for a clinically important benefit (OR 0.75) and extended close to the line of no effect, despite excluding harm.

b. Downgraded one level for imprecision because the pooled estimate was based on a limited number of events and the 95% CI crossed the line of no effect, including both a potentially important reduction in HHF and no clear difference between CSP and BiVP.

c. Downgraded one level for imprecision because the pooled estimate was based on a limited number of deaths and the 95% CI was wide, crossing the line of no effect and including both a potentially important reduction and a possible increase in all-cause mortality with CSP versus BiVP.

d. Downgraded one level for imprecision because the 95% CI was wide and included both a small improvement and a potentially clinically important increase in 6-minute walk distance, leaving uncertainty regarding the magnitude of functional benefit with CSP versus BiVP.

e. Downgraded one level for imprecision because the 95% CI included both a trivial effect and a potentially meaningful improvement in NYHA functional class, limiting certainty regarding the magnitude of symptomatic benefit with CSP versus BiVP.

f. Downgraded one level for imprecision because the 95% CI crossed the line of no effect and included both a potentially small improvement and a potentially small worsening in quality of life with CSP versus BiVP, limiting certainty regarding the treatment effect.

# **Supplemental Table 7**. PRISMA 2020 Checklist

| **Section and Topic** | **Item #** | **Checklist item** | **Location where item**  **is reported** |
| --- | --- | --- | --- |
| **TITLE** | | |  |
| Title | 1 | Identify the report as a systematic review. | Title page, p. 1 |
| **ABSTRACT** | | |  |
| Abstract | 2 | See the PRISMA 2020 for Abstracts checklist. | Abstract, p. 1 |
| **INTRODUCTION** | | |  |
| Rationale | 3 | Describe the rationale for the review in the context of existing knowledge. | Introduction, pp. 3–4 |
| Objectives | 4 | Provide an explicit statement of the objective(s) or question(s) the review addresses. | End of Introduction, p. 4 |
| **METHODS** | | |  |
| Eligibility criteria | 5 | Specify the inclusion and exclusion criteria for the review and how studies were grouped for the syntheses. | Methods, Section 2.2, pp. 4–5; Section 2.7, pp. 6–7 |
| Information sources | 6 | Specify all databases, registers, websites, organisations, reference lists and other sources searched or consulted to identify studies. Specify the date when each source was last searched or consulted. | Methods, Section 2.1, p. 4 |
| Search strategy | 7 | Present the full search strategies for all databases, registers and websites, including any filters and limits used. | Supplemental Tables 1–4 |
| Selection process | 8 | Specify the methods used to decide whether a study met the inclusion criteria of the review, including how many reviewers screened each record and each report retrieved, whether they worked independently, and if applicable, details of automation tools used in the process. | Methods, Section 2.4, p. 5 |
| Data collection process | 9 | Specify the methods used to collect data from reports, including how many reviewers collected data from each report, whether they worked independently, any processes for obtaining or confirming data from study investigators, and if applicable, details of automation tools used in the process. | Methods, Section 2.5, p. 5 |
| Data items | 10a | List and define all outcomes for which data were sought. Specify whether all results that were compatible with each outcome domain in each study were sought (e.g. for all measures, time points, analyses), and if not, the methods used to decide which results to collect. | Methods, Sections 2.3 and 2.5, pp. 5–6 |
|  | 10b | List and define all other variables for which data were sought (e.g. participant and intervention characteristics, funding sources). Describe any assumptions made about any missing or unclear information. | Methods, Section 2.5, p. 5 |
| Study risk of bias assessment | 11 | Specify the methods used to assess risk of bias in the included studies, including details of the tool(s) used, how many reviewers assessed each study and whether they worked independently, and if applicable, details of automation tools used in the process. | Methods, Section 2.6, p. 6 |
| Effect measures | 12 | Specify for each outcome the effect measure(s) (e.g. risk ratio, mean difference) used in the synthesis or presentation of results. | Methods, Section 2.7, p. 6 |
| Synthesis methods | 13a | Describe the processes used to decide which studies were eligible for each synthesis (e.g. tabulating the study intervention characteristics and comparing against the planned groups for each synthesis (item #5)). | Methods, Sections 2.2, 2.3, and 2.7, pp. 4–7 |
|  | 13b | Describe any methods required to prepare the data for presentation or synthesis, such as handling of missing summary statistics, or data conversions. | Methods, Section 2.7, p. 6 |
|  | 13c | Describe any methods used to tabulate or visually display results of individual studies and syntheses. | Methods, Sections 2.5 and 2.7, pp. 5–7 |
|  | 13d | Describe any methods used to synthesize results and provide a rationale for the choice(s). If meta-analysis was performed, describe the model(s), method(s) to identify the presence and extent of statistical heterogeneity, and software package(s) used. | Methods, Section 2.7, pp. 6–7 |
|  | 13e | Describe any methods used to explore possible causes of heterogeneity among study results (e.g. subgroup analysis, meta-regression). | Methods, Section 2.7, p. 7 |
|  | 13f | Describe any sensitivity analyses conducted to assess robustness of the synthesized results. | Methods, Section 2.7, p. 7; Results, Section 3.16, pp. 10–11; Supplemental Figures 6–47 |
| Reporting bias assessment | 14 | Describe any methods used to assess risk of bias due to missing results in a synthesis (arising from reporting biases). | Methods, Section 2.7, p. 7 |
| Certainty assessment | 15 | Describe any methods used to assess certainty (or confidence) in the body of evidence for an outcome. | Methods, Section 2.8, p. 7; Supplemental Table 6 |
| **Section and Topic** | **Item #** | **Checklist item** | **Location where item**  **is reported** |
| **RESULTS** | | |  |
| Study selection | 16a | Describe the results of the search and selection process, from the number of records identified in the search to the number of studies included in the review, ideally using a flow diagram. | Results, Section 3.1, p. 7; Supplemental Figure 1 |
|  | 16b | Cite studies that might appear to meet the inclusion criteria, but which were excluded, and explain why they were excluded. | Supplemental Figure 1 |
| Study characteristics | 17 | Cite each included study and present its characteristics. | Results, Section 3.1, pp. 7–8; Table 1; Supplemental Table 5 |
| Risk of bias in studies | 18 | Present assessments of risk of bias for each included study. | Results, Section 3.1, p. 8; Supplemental Figure 2 |
| Results of individual studies | 19 | For all outcomes, present, for each study: (a) summary statistics for each group (where appropriate) and (b) an effect estimate and its precision (e.g. confidence/credible interval), ideally using structured tables or plots. | Figures 1–3; Supplemental Figures 3–5 |
| Results of syntheses | 20a | For each synthesis, briefly summarise the characteristics and risk of bias among contributing studies. | Results, Sections 3.1–3.15, pp. 7–10 |
|  | 20b | Present results of all statistical syntheses conducted. If meta-analysis was done, present for each the summary estimate and its precision (e.g. confidence/credible interval) and measures of statistical heterogeneity. If comparing groups, describe the direction of the effect. | Results, Sections 3.2–3.15, pp. 8–10; Figures 1–3; Supplemental Figures 3–5 |
|  | 20c | Present results of all investigations of possible causes of heterogeneity among study results. | Results, Sections 3.2–3.15, pp. 8–10; subgroup analyses reported within each outcome section |
|  | 20d | Present results of all sensitivity analyses conducted to assess the robustness of the synthesized results. | Results, Section 3.16, pp. 10–11; Supplemental Figures 6–47 |
| Reporting biases | 21 | Present assessments of risk of bias due to missing results (arising from reporting biases) for each synthesis assessed. | Results, Section 3.17, p. 11; Supplemental Figures 48–54 |
| Certainty of evidence | 22 | Present assessments of certainty (or confidence) in the body of evidence for each outcome assessed. | Results, Section 3.18, p. 11; Supplemental Table 6 |
| **DISCUSSION** | | |  |
| Discussion | 23a | Provide a general interpretation of the results in the context of other evidence. | Discussion, Sections 4.1–4.7, pp. 11–14 |
|  | 23b | Discuss any limitations of the evidence included in the review. | Discussion, Section 4.10, pp. 14–15 |
|  | 23c | Discuss any limitations of the review processes used. | Discussion, Section 4.10, pp. 14–15 |
|  | 23d | Discuss implications of the results for practice, policy, and future research. | Discussion, Sections 4.8–4.9, pp. 14–15; Conclusions, p. 15 |
| **OTHER INFORMATION** | | |  |
| Registration and protocol | 24a | Provide registration information for the review, including register name and registration number, or state that the review was not registered. | Methods, Section 2, p. 4; Protocol statement, p. 15 |
|  | 24b | Indicate where the review protocol can be accessed, or state that a protocol was not prepared. | Methods, Section 2, p. 4; Protocol statement, p. 15 |
|  | 24c | Describe and explain any amendments to information provided at registration or in the protocol. | Methods, Section 2, p. 4: “prespecified analytical framework was followed without deviation” |
| Support | 25 | Describe sources of financial or non-financial support for the review, and the role of the funders or sponsors in the review. | Acknowledgements, p. 15 |
| Competing interests | 26 | Declare any competing interests of review authors. | Conflict of interest, p. 15 |
| Availability of data, code and other materials | 27 | Report which of the following are publicly available and where they can be found: template data collection forms; data extracted from included studies; data used for all analyses; analytic code; any other materials used in the review. | Data availability, p. 15 |

*From:* Page MJ, McKenzie JE, Bossuyt PM, Boutron I, Hoffmann TC, Mulrow CD, et al. The PRISMA 2020 statement: an updated guideline for reporting systematic reviews. BMJ 2021;372:n71. doi: 10.1136/bmj.n71

For more information, visit: <http://www.prisma-statement.org/>
